# Education Research: Defining Competencies and Consensus in Canadian Epilepsy Fellowship Training: A Thematic Discourse Analysis of Expert Perspectives

**DOI:** 10.1212/NE9.0000000000200318

**Published:** 2026-05-19

**Authors:** Anita N. Datta, Kristin M. Ikeda, Aristides Hadjinicolaou, Gianluca D'Onofrio, Julia Jacobs, Juan Pablo Appendino, Carlos Ivan Salazar Cerda, Eliane Kobayashi, Rajesh Ramachandrannair, Kenneth A. Myers, Aylin Y. Reid, Jazlin Ebenezer, Esther Bui

**Affiliations:** 1 Division of Neurology, BC Children's Hospital, University of British Columbia, Vancouver, Canada;; 2 Division of Neurology, The Queen Elizabeth II Health Sciences Centre, Dalhousie University, Halifax, Nova Scotia, Canada;; 3 Division of Neurology, CHU Sainte-Justine, Montréal, Quebec, Canada;; 4 Division of Neurology, Alberta Children's Hospital, University of Calgary, Canada;; 5 Division of Neurology, Jim Pattison Children's Hospital, University of Saskatchewan, Saskatoon, Canada;; 6 Department of Neurology and Neurosurgery, Montreal Neurological Institute, McGill University, Quebec, Canada;; 7 Department of Neurology, Peter O'Donnell Jr. Brain Institute, University of Texas Southwestern Medical Center, Dallas;; 8 Division of Neurology, McMaster Children's Hospital, McMaster University, Hamilton, Ontario, Canada;; 9 Research Institute of the McGill University Health Centre, Montréal, Quebec, Canada;; 10 Division of Neurology, Department of Pediatrics, Montreal Children's Hospital, McGill University, Quebec, Canada;; 11 Krembil Brain Institute, University Health Network, Toronto, Ontario, Canada;; 12 Department of Medicine (Neurology), University of Toronto, Ontario, Canada; and; 13 Teacher Education Division, College of Education, Wayne State University, Detroit, MI.

## Abstract

**Background and Objectives:**

Epilepsy fellowship training in Canada is heterogeneous in program length, extent of electroencephalography (EEG) training, and exposure to transitional and surgical care. This study aimed to develop a national framework for epilepsy fellowship curricula by analyzing expert consensus within the Canadian League Against Epilepsy (CLAE) through thematic discourse analysis.

**Methods:**

We conducted a qualitative thematic discourse analysis of recorded CLAE expert panel meetings with 22 participants, including fellowship program directors and faculty educators. Discussions addressed core competencies, assessment practices, program duration, transition training, and advanced care. Transcripts were analyzed to identify recurrent discursive patterns and areas of alignment and tension.

**Results:**

Six interrelated themes were identified, emphasizing early mastery of foundational competencies, a staged introduction of advanced skills, hybrid time-and-competency structures, the formative use of EEG examinations, flexible transition and surgical exposure pathways, and guidance rather than governance. Participants negotiated national coherence while preserving institutional flexibility.

**Discussion:**

The findings support early acquisition of core competencies, context-sensitive advanced training, and formative assessment guided by competency-based medical education and sociocultural learning principles. This framework balances standardization with institutional flexibility to improve patient safety, trainee preparedness, and equitable access across Canadian programs.

## Introduction

Epilepsy fellowship training in Canada is the only postresidency pathway for advanced training in epilepsy and EEG. However, epilepsy fellowships in Canada are not formally overseen or accredited, and programs are structured as epilepsy fellowships (covering both clinical care and neurophysiology). Prior Delphi consensus work and program analyses have demonstrated substantial heterogeneity across programs in duration, scope, funding models, and clinical exposure, including pediatric-adult transition care positions.^1,2^ Trainees include Canadian neurologists, likely to remain in domestic practice, and international physicians who will apply their training abroad, with career goals ranging from general EEG competence to subspecialized epileptology.^1^ This variability has resulted in inconsistent trainee experiences and underscores the need for evidence-informed national guidance.

Early mastery of core competencies, such as EEG interpretation, patient counseling, and therapeutic decision making, as defined by the International League Against Epilepsy (ILAE) competency framework,^3^ is essential for patient safety and clinical readiness.^4^ However, access to advanced skills (e.g., intracranial EEG, presurgical evaluation) remains uneven and is often constrained by institutional resources.^1,5^ Sociocultural learning theory emphasizes that expertise develops through scaffolded participation in authentic clinical communities, highlighting progression through mentorship and practice rather than passive knowledge transfer.^6-8^

To examine how expert educators negotiate these tensions, we used thematic discourse analysis, which focuses on how meaning, authority, and norms are constructed through interaction.^9,10^ This approach aligns with sociocultural learning theory and competency-based medical education (CBME), both of which emphasize situated, dialogic learning.^11-14^

This study aimed to develop national recommendations for epilepsy fellowship training by analyzing expert discourse within the Canadian League Against Epilepsy (CLAE). We examined how faculty constructed core and advanced competencies, negotiated fellowship duration and assessment practices, and articulated the role and limits of national guidance in the absence of formal accreditation. This qualitative analysis complements a previously published Delphi study that established quantitative agreement on training structure.^1^

## Methods

### Study Design

We conducted a qualitative study using thematic discourse analysis to examine how expert participants constructed meaning, negotiated authority, and justified positions during national discussions on epilepsy fellowship training. Analysis focused on rhetorical and interactional features of language (e.g., hedging, alignment, boundary drawing, reframing) rather than topical frequency alone.^15-18^ This approach is well-suited to medical education research examining the social production of professional norms and educational standards.

### Participants and Data Collection

In October 2021, the CLAE convened a postgraduate epilepsy education working group comprising a pediatric epileptologist, 2 adult epileptologists, and 3 epilepsy fellows. All fellowship program directors in the country and leaders of the Canadian Epilepsy Teaching Network were invited to participate. The epilepsy fellows, selected by the working group for their specific interest in education research, participated in developing the interview guide, formulating discussion questions for panel meetings, and providing feedback on preliminary thematic categories from the trainee perspective.

A total of 22 experts (11 adult, 11 pediatric) participated in 4 recorded virtual meetings held between May 2022 and May 2023 (Table 1). Meetings were 1 hour in duration and followed a structure guided by the working group, with topics reviewed, including the ILAE competency-based curriculum.^3^ Detailed descriptions of these meetings and the Delphi process are provided in the accompanying manuscript.^1^ All participants provided informed consent, and research ethics approval was obtained (University of Toronto REB #42597).

**Table 1:** Demographics of Expert Panel

	N = 22 (%)
Sex	
Male	11 (50)
Female	11 (50)
Age	
25–34	1 (5)
35–44	12 (55)
45–54	6 (27)
55–65	3 (14)
Years of practice	
0–5	3 (14)
6–10	9 (41)
11–15	5 (23)
15-20	2 (10)
>20	3 (14)
Location^a^	
Western	7 (32)
Central	13 (59)
Atlantic	2 (10)
Academic position^b^	
Clinician teacher^c^	13 (59)
Clinician educator^c^	12 (55)
Fellowship director	11(50)
Clinician scientist	5 (23)
Administrator^d^	5 (23)
Residency program director	1 (5)
Other	1 (5)
Fellowship positions	
0	4 (18)
1	8 (36)
2	7 (32)
3	1 (5)
4	1 (5)
5	1 (5)
Funded positions	N = 13
0	4
1	8–9^e^
2	2–5^e^
3	0–1^e^

a Western Canada refers to British Columbia, Alberta, Saskatchewan and Manitoba, Central Canada refers to Ontario and Quebec, Atlantic Canada refers to Newfoundland and Labrador, Nova Scotia, Prince Edward Island, and New Brunswick.

b Some individuals have more than one type of academic position.

c Clinician teacher and Clinician educator refer to formal titles at different Canadian institutions.

d Administrator is a position that involves overseeing the nonteaching, or clinical aspects of an education program.

e A range exists due to variable funding availability.

### Data Collection and Analysis

Audio recordings of panel discussions were transcribed verbatim by a research assistant and imported into NVivo 13 for analysis. Coding combined deductive categories informed by CBME literature (e.g., competencies, assessment, fellowship structure) with inductive identification of emergent discursive patterns.^10,11^ Codes captured both thematic content and recurring rhetorical moves such as legitimating claims, qualifying positions, and delimiting scope. Analysis focused on how participants discursively constructed shared expectations and negotiated areas of tension related to assessment practices, fellowship duration, and advanced training pathways.

Two investigators independently coded all transcripts using an iteratively refined codebook (Table 2), exchanging analytic memos and resolving discrepancies through sustained discussion. A third investigator reviewed coded transcripts, memos, and theme development to ensure interpretive consistency and transparency.

**Table 2:** Qualitative Codebook

Code name	Description	Example quote	Origin (inductive/deductive)
Assessment ambiguity	References to unclear or inconsistent evaluation practices	“We do not really have a standardized way to assess procedural skills across sites.”	Inductive
Gatekeeping through exams	Discourse around the role of certification exams in regulating entry	“The examination is a necessary filter—we cannot just rely on program directors' opinions.”	Inductive
Pediatric-adult divide	Tensions in training across age-based subspecialties	“Adult and pediatric training are worlds apart—we need to acknowledge that.”	Inductive
Core clinical skills	Mentions of essential clinical competencies expected of fellows	“They must be able to independently interpret EEGs by the end of training.”	Deductive
Fellowship structure	Discussions about the organization and duration of training programs	“A one-year fellowship is too short to cover all competencies.”	Deductive
Assessment practices	Comments on methods and tools used for evaluating fellows	“We use workplace-based assessments, but their quality varies.”	Deductive

Table 2 includes the following columns: Code Name—The label assigned to each theme; Description—A brief explanation of what the code captures; Example Quote—A representative excerpt from the data; Origin (Inductive/Deductive)—Whether the code was derived from the data or literature.

This study analyzed anonymized discourse from national expert working groups on epilepsy fellowship education. Consistent with standards for reporting qualitative research, researcher roles, the data collection context, deidentification procedures, and analytic steps are described.^19^ All spoken contributions during the meetings, regardless of later authorship status, were included in the dataset. Quotations are presented solely for illustrative purposes and were fully anonymized to protect confidentiality.

Data were analyzed using reflexive thematic analysis following 6 phases: familiarization, coding, theme development, review, definition, and reporting. Excerpts were selected for their thematic relevance rather than for speaker identity to preserve the integrity of the discourse while maintaining anonymity. As contributors to expert committees may also serve as authors, safeguards were implemented to maintain analytic independence: the coders were not present during meetings, coding occurred without reference to participant identity, reflexivity regarding researcher positionality was documented, and an audit trail was maintained to support analytic rigor.

### Reflexivity Statement

The research team comprised a qualitative methodologist (J.E.) with expertise in science education scholarship, epilepsy fellowship educators with program leadership experience, and 3 current epilepsy fellows. This composition provided diverse perspectives spanning methodological rigor, educational administration, and lived trainee experience. Fellows attended all panel meetings as observers and contributors, offering critical input on trainee needs, practical training challenges, and competency assessment from the learner viewpoint. Throughout analysis, the team engaged in reflexive discussions to surface underlying assumptions, interrogate interpretive choices, and consider alternative readings of the data, recognizing that team members' positions as both insiders and researchers required ongoing critical self-awareness.

### Triangulation and Trustworthiness

Credibility was strengthened through multiple complementary strategies. Analyst triangulation involved independent coding by 2 researchers, with systematic comparison and consensus-building around interpretive decisions. Methodological triangulation examined convergence across data sources, coded discourse segments, reflexive memos, and theme development matrices, requiring themes to be substantiated by evidence across multiple instances rather than isolated cases. A third researcher provided external analytic review, examining the evolution from codes to themes to ensure coherence and grounding in data. This layered approach was further reinforced by maintaining comprehensive audit trails, comparing patterns across panel meetings, and implementing member checking through participant feedback on preliminary findings. Together, these strategies ensured that interpretations were robust, transparent, and well-supported across multiple analytic perspectives.^10,15,17^

### Standard Protocol Approvals, Registrations, and Patient Consents

All participants provided informed consent, and research ethics approval was obtained (University of Toronto REB #42597).

### Data Availability

Anonymized data not published within this article will be made available by request from qualified investigators.

## Results

The results synthesize multimeeting discussions through a thematic discourse-analytic lens, examining how expert interaction constructs professional priorities, boundaries, and consensus. Six integrated themes are presented, each supported by representative excerpts and interpreted in terms of their implications for the implementation of national guidance and fellowship. Table 3 presents how expert participants used recurring discursive features, such as boundary drawing, qualification, and reframing, to negotiate consensus while preserving equity and program legitimacy.

**Table 3:** Thematic-Discourse Features Underpinning Consensus Formation in Epilepsy Fellowship Training

Theme/domain	Discursive feature	Function in expert deliberation	Illustrative example (paraphrased)
Core competencies	Boundary drawing	Establishes shared minimum expectations while distinguishing advanced practice	“Everyone must achieve EEG independence, but not all need surgical exposure.”
Advanced training	Qualification	Limits the scope of consensus in recognition of infrastructural variability	“Only programs with appropriate volumes can support intracranial EEG.”
Fellowship duration	Legitimating through time	Frames time as necessary for identity formation and readiness	“One year allows fellows to function as clinicians, not just observers.”
EEG examination	Reframing	Recasts summative assessment as a formative signal	“The exam guides teaching—it is not a gatekeeping tool.”
Equity across programs	Alignment	Balances standardization with contextual flexibility	“Consensus should guide, not penalize smaller programs.”
Pediatric–adult transition	Problematization	Surfaces structural gaps without mandating uniform solutions	“Transition exposure is important, but must adapt to local workflows.”
National guidance	Authority bounding	Delimits the power of recommendations in the absence of accreditation	“This is guidance, not governance.”

This table illustrates how expert participants used recurring discursive features, such as boundary drawing, qualification, and reframing—to negotiate consensus while preserving equity and program legitimacy.

### Theme 1: From Fragmentation to Foundational Alignment (and Consensus as Dialogic Practice)

Participants are oriented to consensus not as a fixed endpoint but as a negotiated and evolving accomplishment produced through justification, alignment, and recalibration over time. Language functioned to stabilize shared expectations while explicitly preserving programmatic diversity. Across meetings, participants repeatedly invoked a structural absence, the lack of a unified, Canada-wide epilepsy fellowship curriculum, framing it as a shared starting point that justified collective work toward alignment. For example, one participant reflected on the rationale for the project by noting:

There is no standardized epilepsy fellowship curriculum for learning objectives… The objective of this project is to develop consensus-based learning objectives that may lead to standardization across Canada.

In this comment, the impersonal construction (“there is no”) presents the absence of standardization as a structural reality rather than an attributable failure. The subsequent reference to the project's purpose positions the consensus effort as a reasonable response to this gap, while the emphasis on “consensus-based” objectives and the hedged phrasing “may lead to” tempers authority and preserves space for programmatic autonomy.

Participants further grounded variation in concrete geographic terms, transforming diversity into an observable structural fact rather than an evaluative claim.

Ontario has four epilepsy programs: in Quebec, only Montreal, and in the east… only Halifax. The only institutions that do not offer pediatric epilepsy are Hamilton and Halifax.

Here, descriptive enumeration and nonjudgmental language normalize variation as a function of geography and system capacity rather than deficiency, thereby balancing alignment with contextual responsiveness.

This process-oriented view extended to participants' conceptualizations of agreement thresholds during the Delphi process.^1^ Rather than treating the Delphi cutoff as a binary decision point, participants framed it as a heuristic for staging ongoing work.

They’re not all going to be binary yes-or-no… Not reaching a consensus is also an interesting point, and it requires further research and discussions with everyone to determine where we want to go.

Nonconsensus is revalued as meaningful and generative, positioning disagreement as data rather than failure.

We need to identify where there is no consensus and where future work needs to be focused. That is valuable for both the process paper and the consensus statement.

Collectively, these discursive moves construct consensus as developmental rather than final: National alignment is legitimized while remaining bounded by responsiveness to a heterogeneous training landscape. The consensus statement is thus framed as a provisional foundation designed for iterative refinement over time.

### Theme 2: Core Competencies Early; Advanced Competencies Later (Constructing a Tiered, Accessible Competency Structure)

Participants conceptualized competency development as a sequenced, hierarchical process, positioning early mastery of foundational skills as necessary and non-negotiable. Core competencies, defined by the ILAE,^3^ such as basic EEG interpretation, seizure classification, counseling, and treatment decision making, were framed as prerequisites for a safe and legitimate entry into a fellowship program. Convergence emerged not only around *what* fellows should learn, but *when*, normalizing early standardization while deferring specialization.

Everybody thought that all these skills should be accomplished early in training.

The collective phrasing (“everybody thought”) performs consensus work, presenting early mastery of core competencies as self-evident rather than debatable. Foundational skills are thus constructed as universal obligations and baseline expectations for fellowship training.

Participants further drew a boundary between foundational competence and advanced practice, explicitly sequencing complexity over time.

We need to make sure that the core skills are solid before we move into the more complex areas like intracranial EEG or electrical source imaging.

Here, advanced techniques are characterized as “more complex,” thereby justifying delayed exposure and framing progression as linear and protective. This sequencing frames early standardization as necessary for safety while guarding against premature engagement with high-stakes modalities.

At the same time, participants resisted the universalization of advanced competencies by invoking variability in career trajectories and institutional contexts.

Not everyone is going to be doing intracranial EEG or working in a surgical center, so we need to be thoughtful about who needs what.

This formulation normalizes heterogeneity, legitimizes selective depth rather than uniform coverage, and introduces deliberation as a curricular ethic. Advanced competencies are thus repositioned as context-dependent extensions rather than core requirements.

Collectively, these discursive moves aligns with CBME literature, emphasizing early skill acquisition and progressive responsibility.^5,11,12,20^ They also support a tiered competency model in which early, universal skills are framed as essential, while advanced competencies remain flexible, role-specific, and responsive to diverse training contexts.

### Theme 3: Duration, Definitions, and Near-Term Hybrid Models (Feasibility Without Dilution)

Participants engaged in boundary drawing around fellowship duration, using time as a key marker of legitimacy and scope. Duration was repeatedly mobilized to distinguish comprehensive clinical training from narrower experiences, positioning minimum length as central to what can credibly be named a fellowship.

If it is clinical, it’s at least one year… six months is way too short to call it a fellowship.

The emphasis on naming (“to call it”) ties duration to professional meaning and public credibility, framing time not as a logistical detail but as constitutive of the fellowship identity.

This boundary was reinforced through categorical exclusion, with participants explicitly refusing to label shorter programs as epilepsy fellowships.

I wouldn’t call it an epilepsy fellowship… It’s not an epilepsy fellowship.

Repetition and negation stabilize the boundary between full clinical training and more limited skills-based experiences. Importantly, shorter programs were not rejected outright; rather, they were repositioned as legitimate only if transparently differentiated, thereby protecting the epistemic authority of the fellowship designation.

Alongside these firm boundaries, participants introduced pragmatic considerations that softened rigid time-based prescriptions. Rather than treating duration alone as sufficient, they articulated hybrid models combining minimum time requirements with competency-based organization.

A hybrid approach—stipulating minimum time but organized around competencies—seems most workable, with details resolved program by program.

This formulation performs feasibility work, maintaining shared guardrails while shifting implementation to local contexts.

Time was also framed as necessary for exposure to low-frequency, high-stakes clinical experiences, particularly in advanced neurophysiology.

Time is important to ensure exposure to EEG and other low-volume experiences; if the time is shorter, they may not get that exposure.

Here, duration functions as a protective mechanism, linked to structural realities of case mix rather than tradition. At the same time, participants resisted equating time alone with competence, cautioning against extreme applications of time-independent competency logic.

It would be silly if someone came with good EEG skills and we decided after one month that they were ‘done’… Funding and logistics also matter.

Evaluative language delegitimizes reductive interpretations of competency-based training, while references to funding and logistics foreground material constraints as integral to program design.

Participants further normalized discretion and heterogeneity across programs, particularly with respect to 1-year versus 2-year fellowships and variable research expectations.

Maybe this is something that can remain at the discretion of the program… especially given one versus two-year fellowships and the variability in what counts as research.

Flexibility is thus framed as appropriate and inevitable, positioning standardization as negotiated and staged rather than immediately.

Collectively, these discursive moves balance rigor and feasibility: Credential integrity is protected through clear naming and minimum duration, while hybrid time-plus-competency models legitimize pragmatic adaptation to local constraints without diluting standards. This balance aligns with empirical evidence suggesting that extended training is beneficial for complex subspecialties.^5,21^

### Theme 4: Assessment and EEG Examination (Mandatory Participation, No Passing Requirement)

Participants engaged in assessment boundary work by incorporating the Canadian Society of Clinical Neurophysiology (CSCN) EEG examination as a structuring device while resisting its elevation as a defining marker of fellowship competence. Although recognized as the only available external credential, the examination was discursively positioned as limited in scope, focused on routine EEG rather than the full range of clinical reasoning and decision making central to epileptology. This framing allowed participants to endorse the exam's use without redefining fellowship identity.

I think it can only make the education component of a fellowship stronger if everyone who is training those fellows knows it’s important to get them to pass that exam.

Here, the examination is constructed not as a terminal gatekeeping mechanism but as a coordinating signal that aligns teaching priorities and shared expectations among faculty. Emphasis is placed on strengthening the educational environment rather than certifying individual worth.

At the same time, participants explicitly problematized high-stakes uses of the examination, introducing effective and pedagogical consequences as risks of overemphasis.

Many of the fellows got very stressed… if we focus too much on this exam… that actually leads to a decrement in the other parts that we also want to teach in epilepsy.

Stress is framed as a systemic effect rather than an individual shortcoming, legitimizing caution against narrowing fellowship training around a single assessment.

This tension is resolved discursively by separating participation from outcome.

I think it should be mandatory to take the exam, but not mandatory to pass it.

Mandatory participation establishes accountability and comparability across programs, while removing the passing requirement reduces stakes and protects fellows from disproportionate consequences associated with a narrowly scoped test.

Collectively, these discursive moves construct a formative assessment posture: the CSCN EEG examination is positioned to structure teaching, benchmarking, and program-level reflection without determining fellowship completion. This stance preserves curricular breadth, accommodates institutional variability, and ensures that fellowship identity remains anchored in comprehensive clinical training rather than examination performance alone.

### Theme 5: Transition Exposure and Advanced Surgical Pathways (Valued Outcomes, Adaptable Delivery)

Participants identified transition exposure and advanced surgical training as desirable educational outcomes, while deliberately decoupling them from fixed structural requirements. The pediatric-adult transition was repeatedly framed as an important developmental experience, yet participants foregrounded uneven access to formal transition clinics, particularly outside large academic centers. Rather than interpreting this variability as a deficit, participants used it to justify flexible approaches to achieving comparable learning outcomes.

This tension is visible in participants' careful qualification of utility and feasibility. Note the following observation:

For pediatric training that might not be so useful… not all centers have a true transition clinic to begin with.

This statement indicates that the value of certain learning experiences depends on both the trainee's developmental trajectory and the institution's capacity. Transition exposure is thus considered important but not inherently tied to a specific clinic model. At the same time, participants explicitly named the absence of transition experience as a recognized gap. As one participant noted,

We rarely get to see the transition from pediatric to adult care—it’s a gap.

Here, the lack of exposure is framed not as an individual or programmatic failure, but as a structural limitation. This framing legitimizes efforts to address transition learning while simultaneously explaining why uniform provision across programs cannot be assumed.

Participants resolved this tension by endorsing adaptable delivery mechanisms, such as integrated adult rotations for pediatric fellows, rather than mandates tied to specific infrastructures. In this way, exposure to transition concepts is preserved as a shared educational expectation, while delivery remains responsive to local realities.

A parallel discourse emerged around advanced epilepsy and surgical competencies. Participants strongly resisted making these competencies universally mandatory, particularly in programs without surgical infrastructure. This resistance is articulated in the following concern:

I think there are some centers that… don’t do surgery… if we say things like this have to be mandatory, then we’re basically saying their fellowships aren’t real.

Here, rigid standardization is framed as potentially delegitimizing, equating mandatory requirements with the exclusion of otherwise valid programs. Through this framing, advanced surgical competencies are repositioned as later-stage, elective, or specialized pathways rather than defining features of fellowship identity. This positioning allows programs without surgical volume to maintain legitimacy, while enabling centers with capacity to offer advanced depth aligned with trainee goals.

Taken together, these discursive patterns support findings on variability in fellowship experiences across institutions.^5,22^ They also support an outcome-oriented flexibility model in which exposure to transition and presurgical concepts is protected as educationally valuable but delivered through context-sensitive pathways rather than mandated structures. This approach maintains fairness across resource-variable programs while aligning advanced training opportunities with both local capacity and individual career trajectories.

### Theme 6: Guidance, Not Governance (Scope, Authority, and Next Steps)

Participants articulated clear limits on the authority of the consensus statement, engaging in explicit scope calibration to distinguish guidance from governance. In the absence of a national accreditor, the statement was designed to be nonenforceable, emphasizing professional discretion rather than mandate.

We don’t have the authority to impose standards; programs will do what they need to do. This should be considered a good beginning, not a binding requirement.

The language of “beginning” frames the consensus as provisional and developmental rather than prescriptive. Rather than undermining legitimacy, restraint is mobilized as a credibility strategy, inviting adoption through usefulness and trust rather than coercion.

Participants further embedded the statement within a staged process of knowledge building and refinement, explicitly orienting to future expansion.

Publications are planned: an education paper on the process and a national consensus statement. The next stage brings in even more of the fellows' perspectives.

This formulation constructs the initiative as iterative and expandable, positioning learner perspectives as essential to the legitimacy of subsequent revisions. The consensus statement is thus framed not as a final authority, but as an evolving scaffold, strengthened through implementation, feedback, and continued deliberation.

## Discussion (Discursive Analytic Framing)

Our previously published Delphi study reported the quantitative outcomes of the national consensus process, identifying the competencies and training elements that achieved agreement among experts. However, Delphi results alone do not capture the interpretive and interactional processes through which consensus is constructed. This discourse analysis complements those findings by examining how participants framed key challenges in epilepsy fellowship training, how areas of disagreement were negotiated, and how shared priorities emerged through discussion. By illuminating the reasoning and professional values underlying the final recommendations, this analysis provides insight into *how and why* the consensus positions reported in the earlier study were ultimately reached.

Participants depict a field in transition, constructing national alignment while preserving equity across heterogeneous programs, trainee goals, and resources. Across themes, they articulate a coherent logic: early universal mastery of core competencies; elective later-stage depth; hybrid time-plus-competency structures; a formative stance toward the CSCN EEG examination; and guidance rather than governance. Together, these discursive moves legitimate coherence without uniformity and frame consensus as developmental rather than prescriptive.

Findings align with CBME's emphasis on baseline capabilities that enable progressive autonomy, operationalized through the distinction between mandatory core skills (EEG interpretation, seizure classification, counseling, therapeutic decision-making) and optional advanced pathways.^1,11,12,23^ Variable agreement on advanced surgical competencies reflects uneven infrastructure across sites, indicating that individualized progression presumes capacity for authentic workplace-based assessment. Socioculturally, mentorship, graduated responsibility, and staged exposure enact legitimate peripheral participation, supporting integration into communities of practice; where formal transition clinics are absent, context-sensitive alternatives such as integrated adult rotations approximate authentic participation.^6-8^

Requiring examination participation without a passing requirement positions the CSCN EEG examination as a coordinating signal for teaching and accountability while avoiding curricular narrowing. The examination functions formatively at the program level, with workplace-based assessments central to capturing the breadth of epileptology practice. Duration and labeling further protect credential integrity: classifying 6-month EEG-focused experiences as “top-up” training and maintaining a minimum of 1 year for clinical fellowships acknowledge that time in community supports professional identity formation. A near-term hybrid model accommodates funding, visa, volume, and assessment constraints while ensuring adequate exposure. Advanced competencies are framed as elective and context dependent, preserving program legitimacy where surgical infrastructure is limited; equity can be supported through regional collaboration, virtual case forums, simulation, and shared curricula.^1^ With absent of a national accreditor, authority is deliberately bounded, with guidance fostering adoption through usefulness and professional trust.

Findings reflect expert discourse within a consensus process rather than direct observation of training practice. Findings reflect views of a select group of educators and program directors and may not fully capture lived experiences, needs, or challenges faced by trainees across diverse institutional contexts. Uptake may vary in the absence of a national accreditor, and resource disparities will continue to shape feasibility. Future work should integrate a larger number of fellows' perspectives, pilot early core competencies across diverse sites, and expand shared mechanisms for advanced learning to support iterative refinement.

Integrated through CBME and sociocultural lenses, this study outlines an implementable path to national alignment: early universal mastery of core competencies, elective later-stage depth matched to context, hybrid structural models, and formative assessment within a guidance-not-governance framework. This approach advances patient safety, trainee readiness, and equity while supporting continued evolution toward a mature competency-based system.

## Glossary

CBME: competency-based medical education
CLAE: Canadian League Against Epilepsy
CSCN: Canadian Society of Clinical Neurophysiology
ILAE: International League Against Epilepsy
